# Morbidity Assessment in Surgery: Refinement Proposal Based on a Concept of Perioperative Adverse Events

**DOI:** 10.1155/2013/625093

**Published:** 2013-05-16

**Authors:** Airazat M. Kazaryan, Bård I. Røsok, Bjørn Edwin

**Affiliations:** ^1^Interventional Centre, Oslo University Hospital-Rikshospitalet, 0027 Oslo, Norway; ^2^Surgical Department, Skien Hospital, Sykehuset Telemark Hospital Trust, 3710 Skien, Norway; ^3^Institute of Clinical Medicine, Faculty of Medicine, University of Oslo, 0316 Oslo, Norway; ^4^Department of Gastrointestinal Surgery, Oslo University Hospital-Rikshospitalet, 0027 Oslo, Norway

## Abstract

*Background*. Morbidity is a cornerstone assessing surgical treatment; nevertheless surgeons have not reached extensive consensus on this problem. *Methods and Findings*. Clavien, Dindo, and Strasberg with coauthors (1992, 2004, 2009, and 2010) made significant efforts to the standardization of *surgical morbidity* (Clavien-Dindo-Strasberg classification, last revision, the Accordion classification). However, this classification includes only *postoperative complications* and has two principal shortcomings: disregard of *intraoperative events* and confusing terminology. Postoperative events have a major impact on patient well-being. However, intraoperative events should also be recorded and reported even if they do not evidently affect the patient's postoperative well-being. The term *surgical complication* applied in the Clavien-Dindo-Strasberg classification may be regarded as an incident resulting in a complication caused by technical failure of surgery, in contrast to the so-called *medical complications*. Therefore, the term surgical complication contributes to misinterpretation of *perioperative morbidity*. The term *perioperative adverse events* comprising both *intraoperative unfavourable incidents* and postoperative complications could be regarded as better alternative. In 2005, Satava suggested a simple grading to evaluate intraoperative surgical errors. Based on that approach, we have elaborated a 3-grade classification of intraoperative incidents so that it can be used to grade intraoperative events of any type of surgery. Refinements have been made to the Accordion classification of postoperative complications. *Interpretation*. The proposed systematization of perioperative adverse events utilizing the combined application of two appraisal tools, that is, the elaborated classification of intraoperative incidents on the basis of the Satava approach to surgical error evaluation together with the modified Accordion classification of postoperative complication, appears to be an effective tool for comprehensive assessment of surgical outcomes. This concept was validated in regard to various surgical procedures. Broad implementation of this approach will promote the development of surgical science and practice.

## 1. State of the Art

A standard way of reporting treatment outcomes has been a concern for surgeons for decades [1, 2]. It contributes to improved health care quality control and facilitates surgical research. Morbidity rate is a key parameter in the evaluation of any medical intervention [3]. Its assessment is especially important at appraisal of surgical innovations [4]. Recently, Dindo and Clavien [5] and Sokol and Wilson [6] initiated a discussion in the World Journal of Surgery regarding the definition of perioperative morbidity. The surgical community has generally recognized a semantic definition of postoperative complications as “any deviation from the ideal postoperative course that is not inherent in the procedure and does not comprise a failure to cure” as pronounced by Dindo and Clavien [5]. However, the dispute has just partly touched the practical challenges related to registration and systematization of perioperative morbidity. In spite of wide acceptance of the need for a standardized taxonomy, surgeons have not yet come to a comprehensive consensus. Herein we try to contribute to a more complete understanding of the term perioperative morbidity.

In 1992, Clavien and co-authors made a first significant attempt to standardize approaches to assessment of perioperative morbidity [7]. In 2004, this classification was revised by Dindo and co-authors and in 2009 by Strasberg and co-authors [8, 9]. The last revision was named “Accordion Severity Grading System of Surgical Complications” (later referred as the Accordion classification); however it has once again received minor amendments made by Porembka and co-authors [9, 10]. During the last two decades this Clavien-Dindo-Strasberg classification system has received a wide application (the classification has been cited in 2815 publications in accordance with the Google School citation engine report on February 2013) and has enabled comparison of surgical outcomes from different institutions with improved accuracy and therefore has enabled a better communication between surgeons around the world. This standardized approach has improved quality of systematic reviews and multicentre studies in surgery. However, what the Accordion classification (as well as earlier versions of Clavien-Dindo-Strasberg classification) defines as surgical complications are in fact only postoperative complications. Thus this classification has two principal shortages: it disregards intraoperative events and contributes to confusing terminology due to the uncertainty of the term “surgical” [11–13]. 

Both intraoperative and postoperative adverse events occur during patient surgical treatment. It is obvious that events occurring during the postoperative period may have a major impact on the patient well-being. However, significant intraoperative events should be also recorded and reported even if they do not lead to postoperative morbidity or do not impact the postoperative well-being of the patient [14]. In the aviation industry, these events are called a “near miss” and reported and discussed in order to understand how to identify an error and prevent recurrent errors in the future [15]. Similarly, the point of reporting adverse events in surgery includes the primary need to reduce their occurrence [16]. Information about intraoperative events could contribute to refinement of surgical strategy, tactics, and techniques as well as adjustment of medication protocols supporting surgical intervention [17]. This point is especially relevant in the evaluation of new surgical techniques or assessment of the operative progress of junior surgeons. When intraoperative adverse events confer postoperative burden to the patients, these events achieve complication status which could be verified during the postoperative period. However, direct cause-effect relationship between a specific intraoperative event and the subsequent postoperative complication can often be difficult to assess. In fact, many postoperative complications do not relate to any single particular intraoperative adverse event per se, but most often are a consequence of a series of “errors without consequence” which accumulate and ultimately result in a complication. Conversely, intraoperative events do not necessarily lead to adverse postoperative patient status, so the term intraoperative unfavourable incident reflects the nature of this phenomenon. By contrast postoperative complications, whether they are directly associated with earlier intraoperative unfavourable incident or not, have always negative impact on patient well-being.

**Figure 1:**
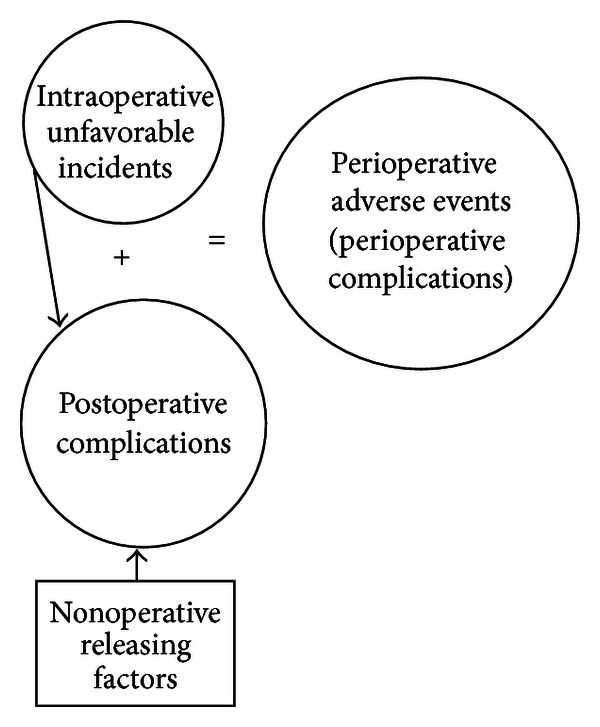
Schematic reproduction of the constituents of perioperative adverse events.

The term surgical complication applied in the Accordion classification may be regarded by many surgeons as an incident resulting in a complication caused by technical failure of the operative intervention, in contrast to the so-called medical complications, but authors of the classification meant the both types of postoperative complications [18–20]. Therefore the term surgical complication will contribute to confusing interpretation of perioperative morbidity and postoperative complications in particular. The term perioperative adverse events comprising both intraoperative unfavourable incidents and postoperative complications could be regarded as better alternative. This term was first applied in the early 1990s in anaesthesiology research [21, 22]. Later on surgeons adapted the term perioperative adverse events. However only since the middle of 2000s use of this term has been expanding in surgical sciences [23–26]. In fact both postoperative complications and intraoperative unfavourable events may be related to either technical surgical or medical (general or anaesthesia related) unfavourable events. The constituents of perioperative adverse events are schematically presented in Figure 1.

We, as many other surgeons, have felt that the best way to report perioperative adverse events by means of combined application of two appraisal tools—the Satava approach to grade intraoperative unfavourable incidents together with the modified Clavien-Dindo-Strasberg classification of postoperative complications [27–31]. However if this concept of morbidity assessment in surgery should be systematically described and accepted worldwide, it will enable surgeons to achieve a new refined and concise standard of reporting of perioperative adverse events. Such an approach to systematization of this concept and refinement of perioperative adverse events reporting is presented herein.

Semantic definitions should also take into account specifics of vocabulary of different languages [32]. The expression perioperative adverse events appears to be more appropriate than *perioperative complications* as it is more comprehensive (including the consequences of both symptomatic postoperative complications and intraoperative unfavourable incidents). We have avoided using the term perioperative morbidity, since morbidity by definition is a term describing illness in an individual, which not necessarily will be the case following an intraoperative unfavourable incident. Thus though the use of the term perioperative adverse events in order to describe both intra- and postoperative unfavourable events are strongly recommended, the term perioperative complications can be used as well.

We elaborated a classification of intraoperative unfavourable incidents on the basis of the Satava approach to surgical error evaluation; it established the ability to grade intraoperative unfavourable incidents to be generalized to any type of surgical intervention (Table 1). We suggest a revision for clarifying and refining the Accordion classification (Table 2).

## 2. Elaboration of the Oslo Classification of Intraoperative Unfavourable Incidents on the Basis of Satava Approach to Surgical Error Evaluation

In 2005, Satava proposed a simple approach to grade surgical errors during operation: Grade I: an error without consequence or near miss; Grade II: an error with immediate identification and correction, also referred to as recovery; Grade III: an error that is unrecognized that leads to a significant consequence or complication [27]. The most critical issue is to develop clear, concise, unambiguous definitions of known intraoperative errors, using quantitative metrics whenever possible. The author primarily used this system to grade errors occurring during simulation of a laparoscopic operation to evaluate the development of surgical skills of trainees. However this approach is suited very well to grade any intraoperative unfavourable incident. We propose the following grading of intraoperative unfavourable incidents on the basis of Satava approach to surgical error evaluation (Table 1).

*Grade I*. Incidents managed without change of operative approach and without expected further consequences for the patient. This includes minor injury of adherent or adjacent organs and minimal change of intraoperative tactics and cases with blood loss over normal range. 

The incident may pass unrecognized or be recognized, but not to be significant enough to result in a postoperative sequelae. These accidents are rare if ever reported and are the equivalent of a “near miss.” 

*Examples *

- Intraoperative bleeding of 700 mL during laparoscopic adrenalectomy in adults.
- Minor small bowel perforation during surgical manipulations at laparoscopic liver resection with immediate closure by endoscopic suturing.
- Small injury to the colon during surgical manipulations of a distal resection of the pancreas with immediate closure by suturing.

The immediate identification of such incidents permits full recovery of the patient.

- Clipping of a lower pole artery during laparoscopic pyeloplasty with no postoperative sequelae.
- Ligating a segmental branch of the right hepatic artery rather than the cystic artery during laparoscopic cholecystectomy, with a resulting minimal elevation of hepatic enzymes but no consequences to the patient.
- Laceration of liver parenchyma during other procedures.
- Thermal injury to the gastric serosa upon division of the short gastric vessels during a laparoscopic fundoplication.

Such cases often pass unrecognized.

*Grade II*. Incidents with expected further consequences for the patient. This includes cases requiring limited resection of intraoperatively injured organs or cases with blood loss which is appreciably over normal range. For laparoscopic/thoracoscopic/endoscopic surgery, it includes intraoperative incidents requiring conversion.

*Examples *

- Intraoperative bleeding of 1200 mL during laparoscopic adrenalectomy managed without conversion.
- Bowel devascularisation during adhesiolysis at laparoscopic liver resection requiring limited bowel resection.
- Conversion to conventional laparotomy to manage intraoperative incident (excluding conversion to limited hand assisted procedures).
- Laceration of the spleen or splenic vessels, necessitating a nonscheduled splenectomy.
- Division of spermatic cord during inguinal dissection resulting in reduced fertility.

While not optimal care, the immediate identification of such errors permits immediate remediation of the error, usually without significant long-term complications but with or without minor near-term consequences.

*Grade III*. Incident leading to significant consequences for patient.

These incidents are often associated with unequivocal errors which are not recognized during surgery; therefore the correction is delayed. Some serious incidents could be recognized during surgery but may be too hard or impossible to manage without significant consequences for patient. Such cases often require postoperative reintervention.

*Examples*

- Any common bile duct injury not recognized until postoperative period.
- Catastrophic intraoperative bleeding which is difficult to manage and which requires major blood transfusions/autotransfusions which can cause postoperative multiorgan failure.
- Injury of renal artery resulting in necessity to remove a healthy kidney during adrenalectomy.
- Stapling of the superior pulmonary vein during inferior lobectomy, requiring pulmonectomy.
- Any bowel injury not recognized until the postoperative period.
- Any type of surgical instrument (sponge, forceps, etc.) left behind in the abdominal cavity following laparotomy.

## 3. Refinement Proposal to the Accordion Classification of Postoperative Complications

First, the original version of the Clavien-Dindo-Strasberg classification included a prolonged postoperative hospital stay as a grading factor, but it was excluded in the revision made by Dindo and co-authors in 2004 [7, 8]. We believe that this factor still represents an important index. Furthermore worldwide tendency to shortening of postoperative stay and clear association between long postoperative stay and postoperative complications have become more prominent [35, 36]. We suggest accounting a doubling of postoperative length-of-stay as grade 1 complication per se if it is caused by medical reasons. For example a postoperative stay twice as long as anticipated due to excessive postoperative chylous ascites after pancreatoduodenectomy should be considered as Grade 1 complication in spite of the fact that usual postoperative ascites is considered an unavoidable sequelae and not a complication. If researchers would use the median value of postoperative stay length at the same institution for that disease and procedure as a reference value, the influence of institution traditions and health care system on severity grading will be largely levelled out.

Second, many surgeons and researches are prone to simplify grading of postoperative complications dividing them in two groups, major and minor [37, 38]. Such classification can be confusing and introduce uncertainty; definitions are required as has been suggested by the somewhat complicated 6-grade system of the Accordion classification. Therefore it would be proper to systemize Grades I–III as minor complications, and Grades IV–VI as major complications. This boundary could be controversial, but the necessity of a reoperation under general anaesthesia is usually associated with major postoperative complications. The formal division to mild (Grade  I), moderate (Grade  II), and severe (Grade III–VI), which is described in the Accordion classification, appears to differ from the subjective perception of practicing surgeons. 

Third, “intervention under general anaesthesia” (as mentioned in the Accordion classification) defining grade  IV could be interpreted differently and therefore requires refinement. The “need for artificially pulmonary ventilation” during patient anaesthesia could be added in parentheses to clarify a boundary. For example, a case where the patient is subject to a postoperative gastroscopy to manage a complication requiring only intravenous patient sedation should be regarded as grade  III; need for intubation would define Grade  IV. 

Fourth, the Accordion classification is argued to be based on a therapy used to treat complication. However the classification does not involve cases when an intervention was done due to suspicion of a postoperative complication. It is quite controversial whether one should consider this event as a complication of a grade corresponding to usual therapy or to not consider such case a complication at all. It may be more appropriate to not consider such events as complications because they may be a result of misinterpretation of patient medical condition. However we recommend mentioning such cases at reporting of perioperative adverse events. For example, a patient who had a liver resection is subject to a diagnostic laparoscopy (under general anaesthesia with intubation) on the 2nd postoperative day for suspicion of bile leakage (peritonism, fever, and leukocytosis) which was not confirmed; the patient recovered uneventful. This case could be regarded as Grade  1 complication due to manifestation of feber.

Fifth, the Accordion classification did not define an exact time period during which one should consider an event occurrence as a postoperative complication. The standard of 30 days (or up to discharge if patient stays longer in the hospital) has received a wide acceptance in the surgical community for *perioperative mortality* and can be regarded as the time frame for inclusion as a postoperative complications as well [39, 40]. The modified classification of postoperative complications are presented in Table 2.

## 4. Conclusion

Despite the tremendous advance of evidence-based medicine, inconsistency in reporting perioperative adverse events is common both in research and hospital records. Severity grading was used in only one-third of large (over 100 patients) randomized controlled studies in surgery from 1990 to 2001 [38]. Moreover all instances represented only a simple division to subcategories of minor and major usually without accurate definition of the boundary between them [37, 38]. Even now many studies are published without appropriate grading of perioperative complications; however in the last few years it has been becoming more common to apply severity grading in surgical research [41, 42]. A considerable number of surgeons are becoming aware of good scientific principles and are applying a severity grading to perioperative morbidity. It seems that in the near future this concept will become a prerequisite of good academic research in the field of surgery. 

New surgical techniques can have a higher rate of perioperative morbidity during their introduction, due to the learning curve. This primarily applies to a higher rate of intraoperative unfavourable incidents rather than to postoperative complication. Pragmatically, the moderately increased perioperative adverse events during introduction of new techniques are being accepted as the consequence of advancing the state of the art of surgery. In essence this is the cost that the surgical community bears for the sake of the further progress in surgery to the benefit of future patients and society as a whole. The new science of medical and surgical simulation is not sophisticated enough yet to ameliorate this cost, and the use of animal training is decreasing due to ethical issues [43, 44]. International legal guidelines and regulations in regard to surgery are in quite an immature state, but this topic has recently received increasing interest [45]. Once a requirement to the unified reporting of perioperative morbidity and acceptability of moderately increased perioperative morbidity for new techniques should be prescribed in international guidelines for good clinical practice.

We admit that in order to get overall picture of outcomes of surgical treatment, researchers should report key outcome parameters in addition to the careful standardized reporting of perioperative adverse events. These parameters can be quite specific in different branches of surgery. Classifications of both intraoperative incidents and postoperative complications are to a large extent universal, covering the needs of mainstream general and gastrointestinal surgery; nevertheless the classification may still have some limitations in the case of application to some specific areas of surgery. In such circumstances, a clarifying modification, which still holds standardized grading principles, could represent a sensible solution and play an important role [46]. However it is not possible to set a threshold for every potential complication in every classification system. That is a shortcoming, but classifications always have had such problems. Either they are too detailed or not detailed enough. The most important feature of a good classification system is to establish quantitative definitions which allow the least possible subjectivity to a researcher to interpret the classification and thus to enable the most objective unified grading of the severity of an intraoperative unfavourable incident or postoperative complication. Application of clear, unambiguous, and user-friendly terminology is a requisite for a good classification.

The proposed approach to systematization of perioperative adverse events utilizing the combined application of two appraisal tools, that is, the elaborated classification of intraoperative unfavourable incidents on basis of the Satava approach to surgical error evaluation together with the modified Accordion classification of postoperative complication, appears to be an effective tool for comprehensive assessment of surgical outcomes. This concept was validated in several studies analysing perioperative adverse events in regard to various procedures in general, gastrointestinal, endocrine, urologic, and paediatric surgery [29–31, 47–53]. We recommend a wide implementation of such approach both in surgical research and practice.

## Figures and Tables

**Table 1:** The proposed classification of intraoperative unfavourable incidents.

Grade	Definition of intraoperative incidents
Grade I	Incidents managed without change of operative approach and without further consequences for the patient. This includes minor injury of adherent or adjacent organs and minimal change of intraoperative tactics and cases with blood loss over normal range*.

Grade II	Incidents with further consequences for the patient This includes cases requiring limited resection of intraoperatively injured organs or cases with blood loss which is appreciably over normal range*. For laparoscopic/thoracoscopic/endoscopic surgery it includes intraoperative incidents requiring conversion.

Grade III	Incident leading to significant consequences for patient.

*Amount of blood loss is known parameter influencing on patient postoperative course and recovery [33, 34]. A normal range of blood loss for each particular procedure is subjective in a certain degree, but one can quantify it in regard to different procedures based both on contemporary scientific literature and values typical for own institution. As example in case of liver resection the values of 1000 mL and 2000 mL can be considered to be within normal range and to be appreciably over normal range, respectively, (corresponding to intraoperative incidents Grades I and II). In case of adrenalectomy the corresponding bounds could be considered as 500 mL and 1000 mL, respectively. While reporting *intraoperative  unfavourable  incidents*, one should indicate this defined bound.

**Table 2:** The refinement proposal to the Accordion classification of postoperative complications [10] (text marked by the italic type presents the modified points in the classification).

Grade^a^	Definition of postoperative complication
Grade I	Requires only minor invasive procedures that can be done at the bedside, such as insertion of intravenous lines, urinary catheters, and nasogastric tubes, and drainage of wound infections. Physiotherapy and antiemetics, antipyretics, analgesics, diuretics, electrolytes, and physiotherapy are permitted. *It includes cases requiring a doubly prolonged postoperative stay* ^b^ * to treat conditions which otherwise are considered as sequel *

Grade II	Requires pharmacologic treatment with drugs other than such allowed for minor complications, for example, antibiotics. *Postoperative *blood transfusions and total parenteral nutrition are also included

Grade III	No general anaesthesia: requires management by an endoscopic, interventional procedure or reoperation without general anaesthesia^c,d^

Grade IV	General anesthesia or single-organ failure

Grade V	General anesthesia and single organ failure or multisystem organ failure (>2 organ systems)

Grade VI	Death *within 30 postoperative days or up to discharge if patient stays longer in the hospital. *

^a^Minor complications: Grade I–III; major complications: Grade IV–VI.

^b^Duration of median hospital stay for that disease and procedure which is present in the particular institution is to be applied as a reference value.

^c^Need for artificially pulmonary ventilation during patient anaesthesia is a boundary to define general anaesthesia.

^d^Cases when an intervention was done due to suspicion of complication (without its confirmation) are not to be regarded as a basis for severity grading. However such cases should be reported (see examples in the text).
